# An efficient framework for estimation of muscle fiber orientation using ultrasonography

**DOI:** 10.1186/1475-925X-12-98

**Published:** 2013-09-30

**Authors:** Shan Ling, Bin Chen, Yongjin Zhou, Wan-Zhang Yang, Yu-Qian Zhao, Lei Wang, Yong-Ping Zheng

**Affiliations:** 1 School of Geosciences and Info-Physics, Central South University, Changsha, China; 2 The Shenzhen Key Laboratory for Low-cost Healthcare, Shenzhen, China; 3 Shenzhen Institutes of Advanced Technology, Chinese Academy of Sciences, Shenzhen, China; 4 Harbin Institute of Technology Shenzhen Graduate School, Shenzhen, China; 5 Interdisciplinary Division of Biomedical Engineering, The Hong Kong Polytechnic University, Hong Kong, China; 6 Affiliated Nanshan Hospital of Guangdong Medical College, Shenzhen, China

**Keywords:** Ultrasound, Muscle, Hough transform, Orientation, Line detection, Image segmentation

## Abstract

**Background:**

Muscle fiber orientation (MFO) is an important parameter related to musculoskeletal functions. The traditional manual method for MFO estimation in sonograms was labor-intensive. The automatic methods proposed in recent years also involved voting procedures which were computationally expensive.

**Methods:**

In this paper, we proposed a new framework to efficiently estimate MFO in sonograms. We firstly employed Multi-scale Vessel Enhancement Filtering (MVEF) to enhance fascicles in the sonograms and then the enhanced images were binarized. Finally, line-shaped patterns in the binary map were detected one by one, according to their shape properties. Specifically speaking, for the long-and-thinner regions, the orientation of the targeted muscle fibre was directly computed, without voting procedures, as the orientation of the ellipse that had the same normalized second central moments as the region. For other cases, the Hough voting procedure might be employed for orientation estimation. The performance of the algorithm was evaluated using four various group of sonograms, which are a dataset used in previous reports, 33 sonograms of gastrocnemius from 11 young healthy subjects, one sonogram sequence including 200 frames from a subject and 256 frames from an aged subject with cerebral infarction respectively.

**Results:**

It was demonstrated in the experiments that measurements of the proposed method agreed well with those of the manual method and achieved much more efficiency than the previous Re-voting Hough Transform (RVHT) algorithm.

**Conclusions:**

Results of the experiments suggested that, without compromising the accuracy, in the proposed framework the previous orientation estimation algorithm was accelerated by reduction of its dependence on voting procedures.

## Background

Ultrasonography is being widely used as a clinical and research tool for dynamic studies of the muscle during contraction and relaxation, since it’s real-time, widely available, radiation-free and low-cost. Muscle architectural characteristics, such as pennation angle or fiber orientation [1-8], fascicle length [1,2,9-12], fascicle curvature [13-16] and muscle thickness [9,17-21], can be extracted from ultrasonography to evaluate the muscle function and activity. And changes of these architectural parameters over the time can form quantitative observations of muscle behavior under contraction.

Since orientation of muscle fiber or aponeuroses is often computed first and then acted as basis of the estimation of muscle fiber length during morphological studies of skeletal muscles [22-24], an efficient realization of automatic measurement of muscle fiber orientation (MFO) would also contribute to the estimation of muscle fiber length which is a significant measure of muscle properties.

Traditionally, the fibers and their orientations in musculoskeletal sonograms were detected manually by drawing lines using NIH image software (National Institutes of Health, Bethesda, MD, USA) (for example, [9,25,26]). Being labor-intensive, the manual method affected the real-time and quantitative observations on muscle behaviors. Therefore, recently the method for automatic estimation of fiber orientation in musculoskeletal sonograms had drawn some attentions [3-8]. Although some methods have been developed to extract curve features of muscle fiber [12,27], the detection of line-shaped patterns in musculoskeletal sonograms, as key step of pennation angle estimation, is still of great interests to the field [9,28-30].

However, automatic methods previously reported were all build on the voting procedures using either Hough [3,5] or Radon [6,7] transform which are computationally expensive. For example, in a previous study, we proposed the Re-voting Hough Transform (RVHT) algorithm to realize the automatic estimation of MFO in muscular sonograms [3]. Because of its sensitivity to noises, we later complemented RVHT by employing Gabor Filtering as a pre-processing procedure to enhance the longitudinal sonograms of fiber [5]. Recently, after the evaluation of Gabor Filtering and another enhancement method, namely multiscale vessel enhancement filtering (MVEF), we found MVEF a better choice with comparable enhancement performance but less computation than Gabor Filtering [20]. Certainly, the involvement of the image enhancement procedures would cost more computation.

In the current study, we propose a new framework for MFO estimation in sonograms by skipping the voting step as much as possible. Instead, the shape properties of the interested regions will be used to output the orientation in most cases, detailed in the method section. Compared with the single RVHT method, this framework is expected to achieve higher efficiency at line angle estimation without compromising the accuracy.

## Methods

The efficient framework for estimation of MFO includes three steps: 1) image enhancement, 2) extraction of object regions, and 3) estimation of MFO.

### Image enhancement using MVEF algorithm

Sonograms are usually affected by speckle noises, which hinder the analysis of musculoskeletal geometry. Taking into account the fact that the fiber in sonograms are tubular and include coherent orientation tendencies, we apply MVEF to enhance the sonograms before the line angle detection. The MVEF method is based on the second order local structure, with excellent noise and background suppression performance [31]. The method includes three steps: the Hessian matrix estimation (including the choice of Gaussian kernels), computation of eigenvector for each scale and processing for the maximum vesselness response. More details can be found in [31].

### Extraction of object regions

In this study, objects to be extracted from sonograms are regions which may represent long and thin muscle fibers. Since fibers, both aponeuroses and other fascia structures, have higher intensity than the background in a sonogram, a straightforward approach to find the potential regions of fibers is to apply a threshold on the enhanced image, so that the pixels whose value is greater than the threshold are regarded as the pixels located in the potential object regions. In this paper, the Otsu’s method is employed to get the optimal threshold [32]. This will result in a binary map, *I*_*map*_, where the white components represent the candidate regions for muscle fibers.

### Estimation of MFO

According to our observations, shapes of the interested object regions could vary and be divided into three different patterns. RA: regions which are long and thin, where each of them represents one major muscle fiber. RB: regions which are long but have branches because of adherence of two or several muscle fibers. RC: regions which are short and possibly from a 'broken’ line caused by partial imaging one single muscle fiber.

Orientations of RA and RC would be calculated as the angle between the y-axis and major axis of ellipse that has the same normalized second central moments as the region. Angles of RB would be calculated using Hough transform (HT).

Specifically speaking, 3 shape measures for the region, aspect ratio *Ar*, width *ω* and length *L*, will be used for classification of RA, RB and RC. In this study, *L* and *ω* are calculated respectively as the length of the major and minor axis of the ellipse that has the same normalized second central moments as the region, and the aspect ratio is defined as *L/ω*.

**Figure 1:**
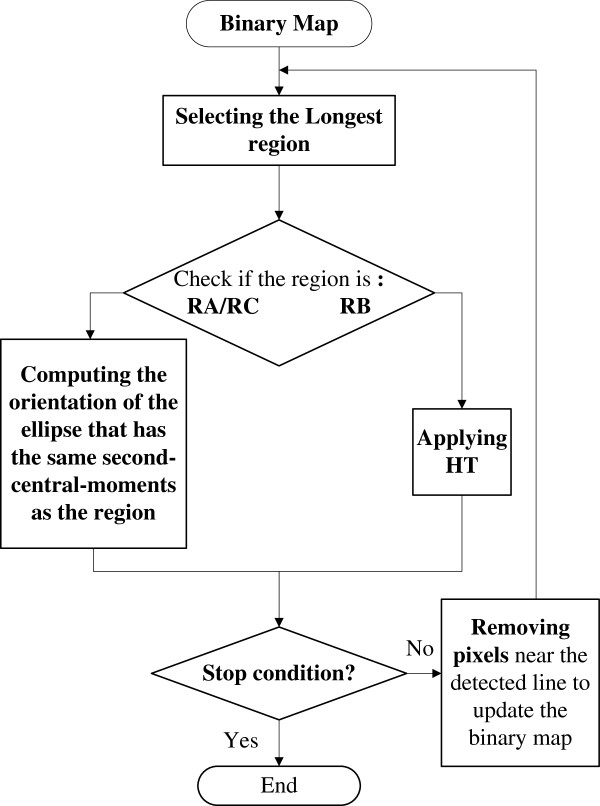
The diagram of the efficient framework for estimating MFO.

The procedures of the proposed framework for MFO estimation on the binary map are shown in Figure 1.

The proposed framework detects lines one by one in *I*_*map*_, starting with the longest region and detailed steps are:

Step 1.  Setting parameters *T*_*1*_, *T*_*2*_, *T*_*3*_ and *N*; *n =* 1; $I_{\mathrm{map}}^{n}=I_{\mathrm{map}}$. (*T*_*1*_, *T*_*2*_ are thresholds for shape measurements *Ar* and *ω* respectively. *T*_*3*_ is the ratio of the length of the last and first detected lines. *N* is the upper limit of line number for each image).

Step 2.  Extracting the longest region and calculating its aspect ratio *Ar*^*n*^, width *ω*^*n*^ and length *L*^*n*^.

Step 3.  If *Ar*^*n*^ > *T*_*1*_ and *ω*^*n*^ < *T*_*2*_, the orientation of the region is estimated as the angle between the *y*-axis and major axis of ellipse that has the same normalized second central moments as the region; Otherwise, applying HT on the region, and the line that with global maximum in the accumulator array is detected.

Step 4.  Removing pixels close to the line detected in step #3 and getting the updated map $I_{\mathrm{map}}^{n+1}$. (This step can remove noises near the line and avoid the duplication in the angle measurement of RC).

Step 5.  Check whether *n* = N or *L*^*n*^ < *L*^*1*^× *T*_*3*_. If not, *n = n +* 1 and repeat from step #2.

### Experiments

#### Experiment setup for normal subjects

The proposed framework is first evaluated using the dataset from previous reports [3,5], which were acquired on biceps and forearm muscles during various typical exercise tasks, from three healthy adult male volunteers, and the detailed experiment setup can be found in [3,5].

Then to further evaluate the proposed framework on same muscle but from different subjects, we designed an experiment example on the sonograms of gastrocnemius. Eleven healthy male subjects (mean ± SD, age = 29.4 ± 1.8 years; body weight 65.9 ± 9.3 kg; height = 170.3 ± 5.1 cm) volunteered to participate in this experiment. No participant had a history of neuromuscular disorders, and all were aware of experimental purposes and procedures. The human subject ethical approval was obtained from the relevant committee in the Hong Kong Polytechnic University, Hung Hum, Hong Kong and informed consents were obtained from subjects prior to the experiment.

The testing position of the subject was in accordance with the Users Guide of a Norm dynamometer (Humac/Norm Testing and Rehabilitation System, Computer Sports Medicine, Inc., Massachusetts, USA). Each subject was required to put forth his maximal effort of isometric plantar flexion for a period of 3 seconds with verbal encouragement provided. The maximal voluntary contraction (MVC) was defined as the highest value of torque recorded during the entire isometric contraction. A rest of 5 min was allowed before the subject performing another MVC test. The MVC torque was then calculated by averaging the two recorded highest torque values from the two tests. The subject was instructed to generate a torque waveform in rough sinusoid shape, up to 90% of his MVC, using ankle plantar flexion movements in prone position. The torque was measured by the aforementioned dynamometer and the reason for choosing 90% MVC as the highest value was to avoid muscle fatigue.

**Figure 2:**
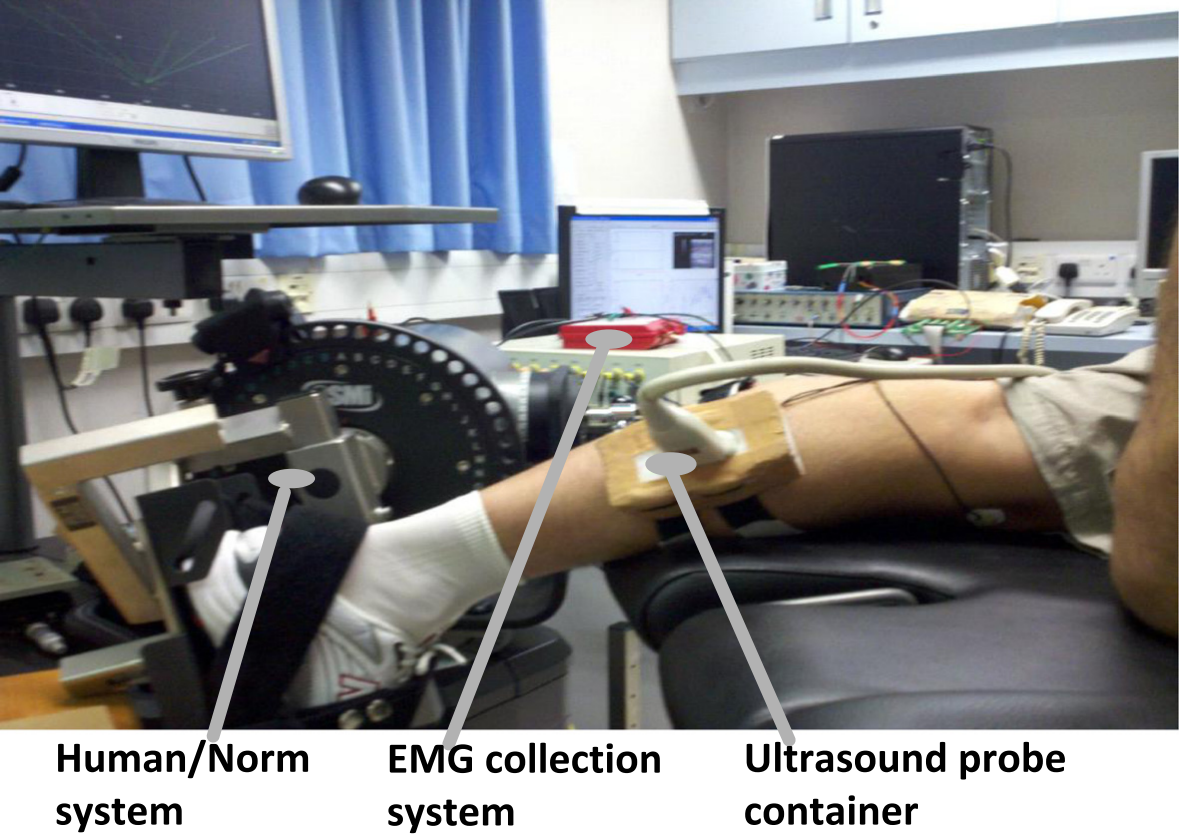
Experimental setup for collecting the torque, EMG and sonograms from the subject’s gastrocnemius during isometric knee extension.

A real-time B-mode ultrasonic scanner (EUB-8500, Hitachi Medical Corporation, Tokyo, Japan) with a 10 MHz electronic linear array probe (L53L, Hitachi Medical Corporation, Tokyo, Japan) was used to obtain ultrasound images of muscles. The long axis of the ultrasound probe was arranged parallel to the long axis of the gastrocnemius and on its muscle belly. The ultrasound probe was fixed by a custom-designed foam container with fixing straps, and a very generous amount of ultrasound gel was applied to secure acoustic coupling between the probe and skin during muscle contractions, as shown in Figure 2. The probe was adjusted to optimize the contrast of muscle fascicles in ultrasound images. Then the B-mode ultrasound images were digitized by a video card (NI PCI-1411, National Instruments, Austin, USA) at a rate of 25 frame/s for later analysis.

Surface electromyography (EMG) signals were collected from the gastrocnemius muscle using bipolar Ag-AgCl electrodes (Axon System, Inc., NY, USA), amplified by a multiple channel amplifier (RM6280 Multi-Channel Biosignal Collection and Processing System, Chengdu Instrument Company, Chengdu, China), with a gain of 2000, filtered separately by 10–400 Hz, 5–100 Hz band-pass analog filters within the amplifier, and then digitized by a 12-bit data acquisition card (NI-DAQ 6024E, National Instruments Corporation, Austin, TX, USA) with a sampling rate of 1 kHz. Ultrasound image sequences, surface EMG and torque signals were simultaneously collected and stored by a custom-made program for ultrasonic measurement of motion and elasticity (UMME, http://www.tups.org).

Totally eleven sequences of gastrocnemius ultrasound images were acquired and each includes 200 images. For each sequence, 3 frames corresponding to the torque at 0%, 45% and 90% MVC were used to evaluate the proposed framework.

Furthermore, one sequence including all 200 frames from one representative subject is used to evaluate the performance of MFO tracking, following the practice of [4,7].

#### Experiment setup for an aged subject with cerebral infarction

**Figure 3:**
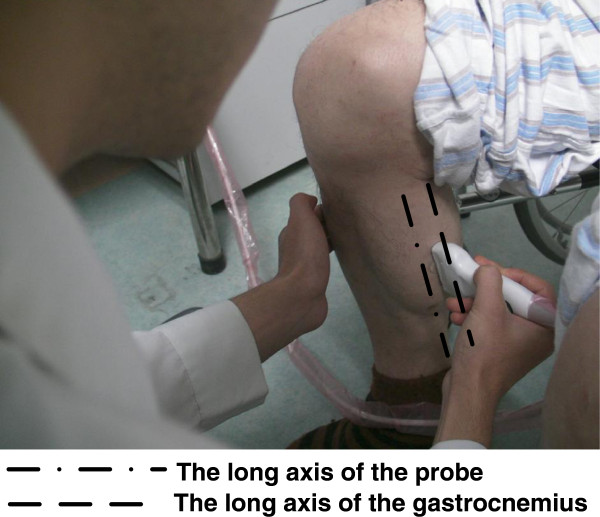
Experimental setup for collecting gastrocnemius sonograms from an aged subject with cerebral infarction.

We also tried to preliminarily evaluate the performance of the proposed framework on sonograms from other than healthy or young subjects. One male subject with unilateral limb dysfunction caused by cerebral infarction (age = 68 years; body weight = 71 kg; height = 1.72 m; right leg dysfunctional) volunteered to participate in this study. The human subject ethical approval was obtained from the relevant committee in Zhujiang Hospital, Guangzhou, China before carrying out the experiment. The subject was briefed about the procedure of experiment and written consents were collected prior to the experiment. The subject was seated with both right hip and knee angles of 90. During measurement, the subject was asked to perform plantar flextion both in left leg (normal) and in right leg (dysfunctional) with his best efforts, and the rough contraction time is about .4 seconds in one exercise. A laptop ultrasound system (SS-10, Sonostar Technologies Co., Limited, Guangzhou, China), with a 7.5 MHz electronic linear array probe, was used to obtain the ultrasonic image sequences. The long axis of the probe was arranged parallel to the long axis of the gastrocnemius muscle (as shown in Figure 3) and during the muscle contraction the probe was managed to keep on well coupled to the muscle belly with a luxury usage of gel by an experienced operator. 128 consecutive frames from each leg were captured. The caregiver of the patient had the SonoStar scanner available only, and they allowed no third-party scanner used inside their medical facilities.

#### Data processing

All images were cropped to remove the imaging tags and retained only the image content, and then processed using the procedures described above. All codes were written in Matlab R2010a.

Five parameters could be controlled by the users. In our experiment, *T*_*1*_, *T*_*2*_ were set to 5, 30 pixels respectively. The removal width, which indicated the number of neighboring pixels to be removed along the line after a line was detected, was set to 17 pixels.

Last-to-first ratio *T*_*3*_ was set to 10%. This ratio together with *N* (the number of lines to be detected for each image) was used as iteration termination conditions. For evaluation purpose in this article, we supposed there would be at most seven representative and interesting linear patterns visible per sonogram from the normal subjects, i.e., patterns corresponding to the skin, bone, superficial aponeurosis, deep aponeurosis and fascicles in between them. Therefore, for normal subjects in this study, we set N, the maximum line per sonogram, to 7.

## Results

### Results on the dataset from previous reports

Totally 314 lines were detected in the 45 sonograms from previous reports [3,5], among which 313 were regarded as being valid according to visual verification and 8 lines were detected using HT.

**Figure 4:**
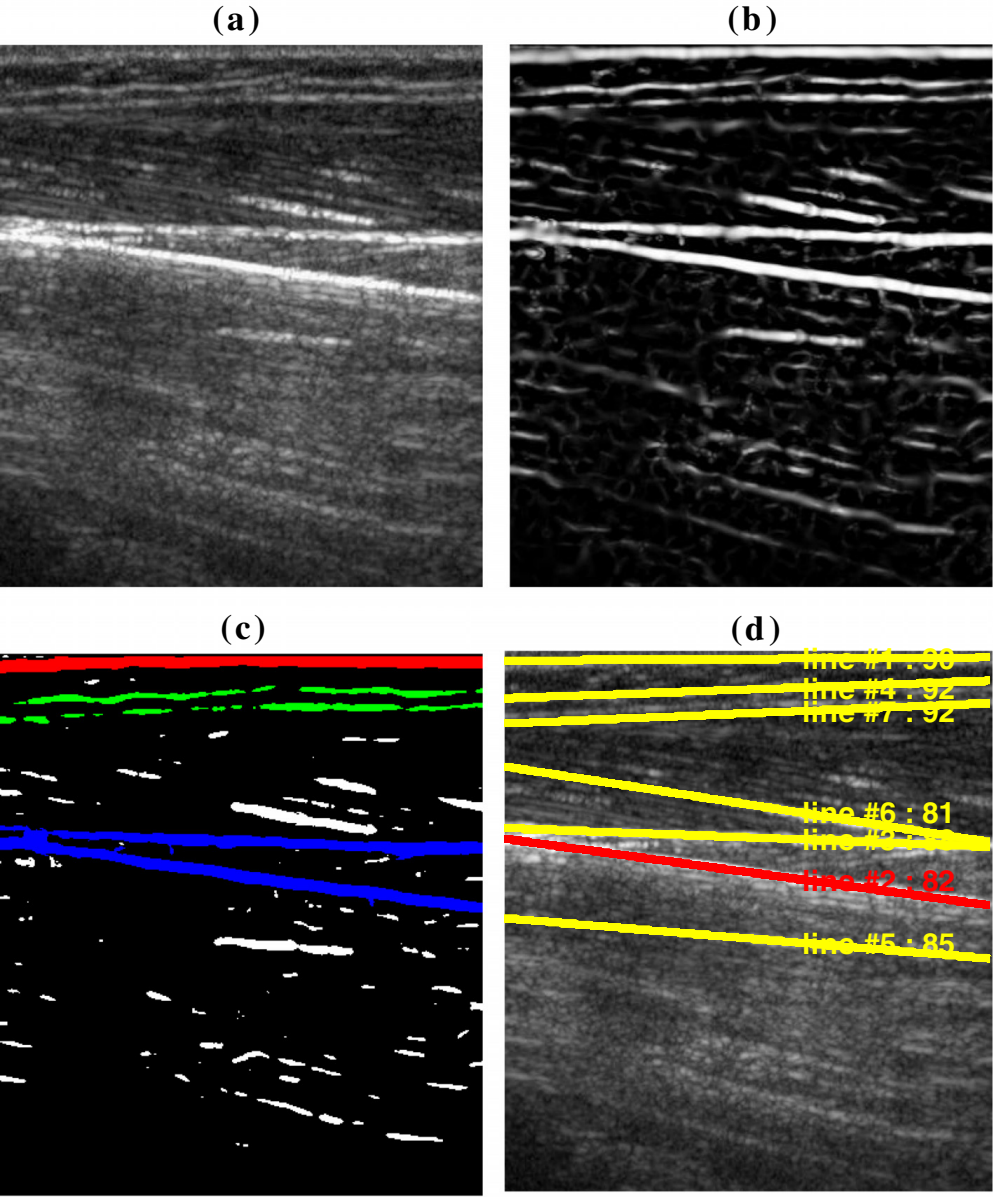
**A representative image of biceps from the previous reports [**[3,5]**] and corresponding MFO results. (a)** The original image. **(b)** The image after MVEF. **(c)** The image after binarization, where red, blue and green regions denote the typical RA, RB and RC respectively. **(d)** MFO detection results, where the angle of red line was detected using HT and the angle of yellow line was estimated as the orientation of the ellipse that has the same second-moments as the region.

A typical original sonogram of biceps and the corresponding results obtained using the proposed method, are shown in Figure 4. The lines and their angle values were marked with the order in which they got detected. MFO here was computed as the angle between the detected line and the vector pointing from the up-left to bottom-left corner of the image, which was the same as the definition of the NIH Image (National Institutes of Health, USA) software.

**Figure 5:**
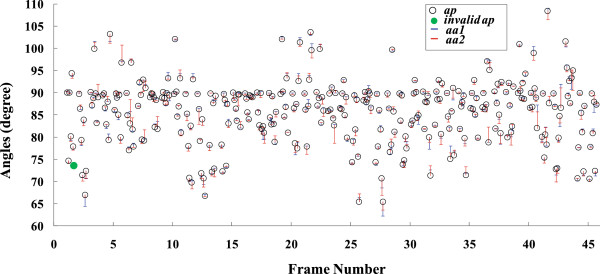
**Comparison of angle estimation results obtained using the proposed and manual methods on the dataset from previous reports [**[3,5]**] where *aa1 aa2 *and *ap *stand for the results from operator #1, #2 and those using the proposed method.**

In order to verify the validity of the method, two operators who were experienced in ultrasound imaging of muscles, were employed to draw lines manually on the original images at locations where lines were detected using the proposed framework, and read the drawn angles using NIH Image software. As a result, 313 lines were marked out manually, all included in the 314 lines detected by the proposed framework. The angles estimated by our method, operator #1 and operator #2 were defined as *ap, aa*1 and *aa*2 respectively and displayed in Figure 5.

**Figure 6:**
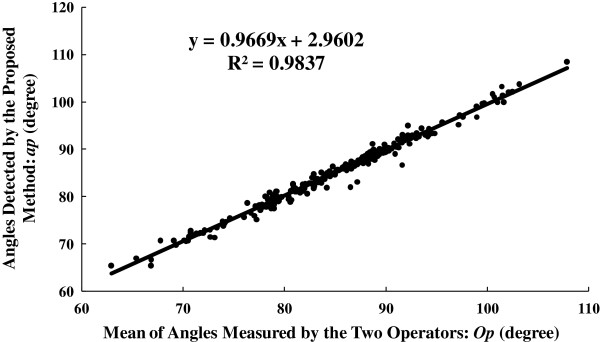
**The correlation between angle estimation results obtained using the proposed and manual methods on the dataset from previous reports [**[3]**,**[5]**].** R is the Pearson product moment correlation coefficient.

**Figure 7:**
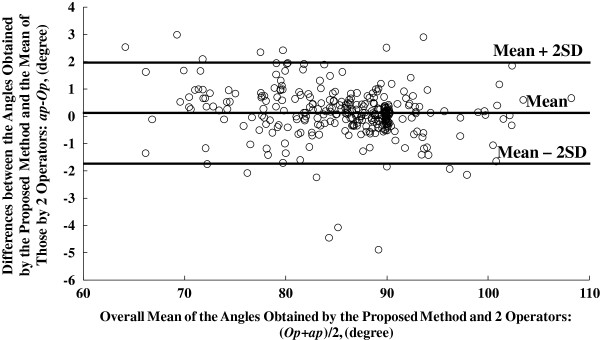
**The Bland-Altman plot of angle estimation results on the dataset from previous reports**[3,5]**.** The units for both x-axis and y-axis are in degrees, where *Op* and *ap* stand for the mean results of the two operators and the results obtained using the proposed method respectively.

To investigate how well our results fit the manually drawn values, the mean of *aa*1 and *aa*2, named as “*Op*”, was compared with the corresponding value of *ap*. The correlation analysis of *Op* and *ap* is shown in Figure 6. Further Bland-Altman plot [33] illustrated in Figure 7 presents the details of the angle measurements statistics. The angle differences between the manual and automatic measurements is 0.11 ± 1.80 at the 95% confidence level.

The general detection performances of the proposed method and RVHT are shown in Table 1, where “RVHT with enhancement” represents RVHT using Gabor filtering as a preprocessing procedure. Comparison of computation cost between the proposed framework and RVHT is displayed in Table 2. We also tested the computing cost of a single Hough Transform and a single procedure of computing the orientation of the ellipse in C (Microsoft Visual Studio 2010, Microsoft Corporation, Seattle, USA), and for a typical edge map with size 390 × 443 and 2055 white pixels in the region of interest, the cost is 149 and 4 seconds respectively for 10000 iterations.

**Table 1:** The comparison of the differences of the angle estimation results (degree)

ap-op**	**Proposed method**	RVHT* **without enhancement**	**RVHT with enhancement**
Mean	0.11	0.18	0.13
Standard error	0.06	0.10	0.07
Median	0.13	0.07	0.08
Standard deviation	0.92	1.23	0.91
Kurtosis	4.03	5.81	13.45
Skewness	-0.52	0.88	-1.57
Range	7.81	10.71	9.41
Minimum	-4.89	-4.90	-5.46
Maximum	2.99	5.81	3.96
Sum	37.40	30.12	24.09
Count (total line detected)	314	168	305
False line detected	1	3	1

*RVHT = revoting Hough transform.

**ap and *op* stand for the results of the proposed methods and the mean of the results from two operators.

**Table 2:** Comparison of computation cost

**Methods**	**Time per-line (s/line)**	**Line detected using HT**	**Total line detected**
Proposed ^a^	0.036	8	314
RVHT^b^	0.142	168	168
RVHT2^c^	0.152	291	291

^a^Proposed: the proposed framework.

^b^RVHT: revoting Hough transform.

^c^RVHT2: RVHT using MVEF as a preprocessing procedure.

### Results on sonograms of gastrocnemius from 11 subjects

**Figure 8:**
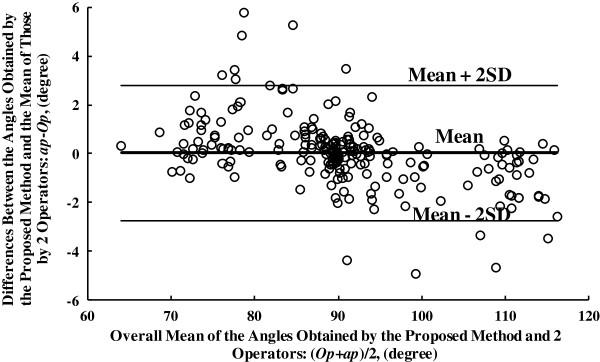
**The Bland-Altman plot of angle estimation results of 33 gastrocnemius sonograms from 11 normal subjects.** The units for both x-axis and y-axis are in degrees, where *Op* and *ap* stand for the mean results of the two operators and the results obtained using the proposed method respectively.

Totally 231 lines were detected in the 33 sonograms of gastrocnemius from 11 normal subjects, among which 217 were regarded as being valid according to visual verification and only 1 line was detected using HT. Blamd-Altman plot of angle detection results for these data, obtained by the proposed framework and the 2 operators, is displayed in Figure 8. The angle differences between the manual and automatic measurements is 0.03 ± 2.72 at the 95% confidence level.

### Results on MFO tracking from 200 frames of one representative subject

**Figure 9:**
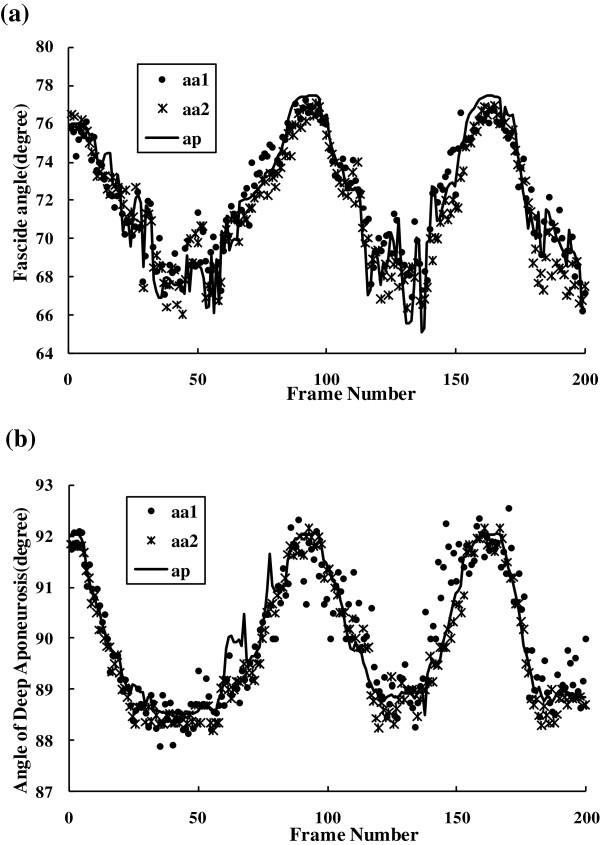
**Comparison of orientations estimated for a dominant fascicle and the deep aponeurosis using manual and proposed methods respectively, where *aa1*, *aa2 *and *ap *stand for the results from operator #1, #2 and the proposed method. (a)** Fascicle orientation estimated by the proposed framework and the two operators. **(b)** Orientation of deep aponeurosis estimated by the proposed framework and the two operators.

**Figure 10:**
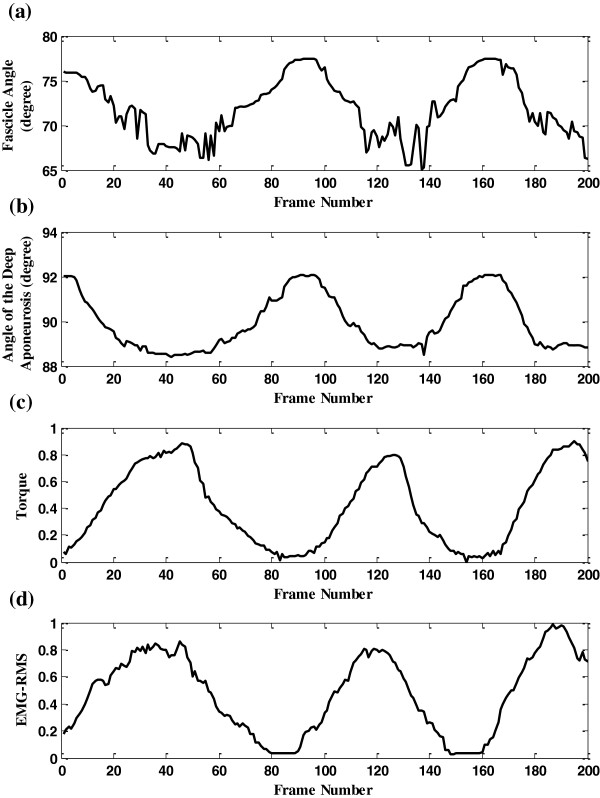
**Orientations of the fascicle and the deep aponeurosis along with the torque and EMG signals. (a)** The signal about the fascicle orientation estimated using the proposed framework, **(b)** the signal about the orientation of the deep aponeurosis estimated using the proposed framework, **(c)** the normalized torque signal, and **(c)** the normalized EMG RMS signal.

We also tracked the orientation changes of a selected dominant fascicle and the deep aponeurosis in a sequence. Comparisons of these two orientations estimated using the proposed and manual methods were shown in Figure 9. It was observed that there were good correlations among the results obtained by the two operators and the proposed framework. Angles of the selected fasicle and the deep aponeurosis were also shown in Figure 10(a-b) together with the corresponding normalized torque signal and the normalized root mean square (RMS, 256-points) values of EMG signal in Figure 10(c-d).

### Results on sonograms from the aged subject with cerebral infarction

**Figure 11:**
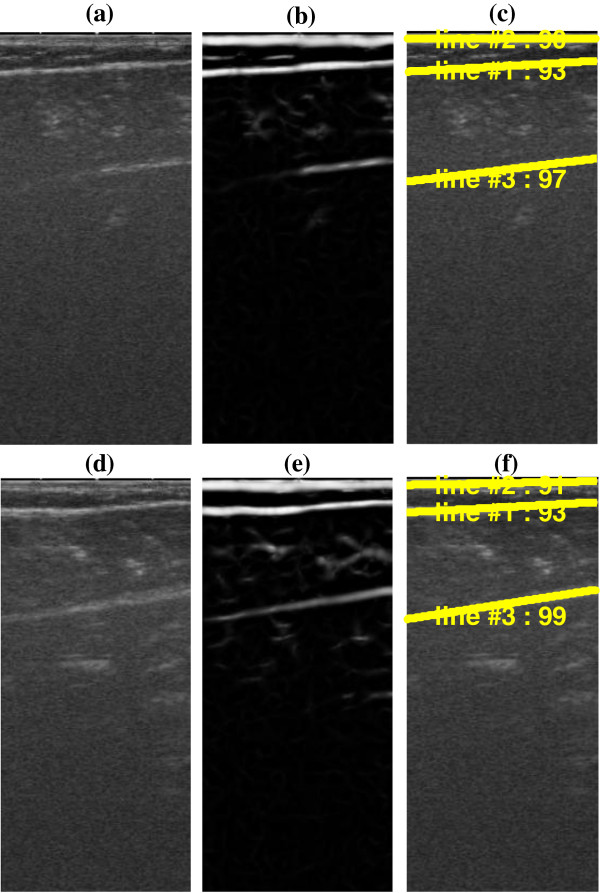
**Results on a patient with unilateral limb dysfunction caused by cerebral infarction. (a)**-**(c)** are a typical frame, its MVEF result and line detection result for left leg respectively. **(d)**-**(f)** are a typical frame, the enhanced image using MVEF and line detection result for right leg (dysfunctional).

Two typical frames from left and right legs of the aged subject and their line detection results estimated using the proposed method are shown in Figure 11. Based on the triceps surae architecture, the maximum line per sonogram *N* was set to 4 for the data from aged subject. 440 and 452 lines were detected for frames for normal and dysfunctional limbs respectively, and among which 3 was invalid according to visual verification. However, totally 67 interested muscle fibers were not detected by the proposed method.

## Discussion

### The effects of the parameters on the line detection

Results of the method are affected by at least 4 parameters, i.e., *T*_*1*_, *T*_*2*_, *T*_*3*_ and *N*.

The total number of detected lines in a sonogram could be determined by *T*_*3*_ and *N.* The maximum line per sonogram *N* could be easily tuned up, if necessary, to accommodate the detection of more localized linear patterns. Taking 45 sonograms of biceps and forearm as examples, if we set *N* to 9 instead of 7, the number of total detected lines would increase from 314 to 378, while the number of false lines not actually corresponding to any one single muscle fiber increased from 1 to 4.

The last-to-first ratio *T*_*3*_ was set to exclude very short regions which may be noises, artefacts or the shorter regions of the broken fiber (whose angles could be the duplications of the longest segment from the same broken fiber). It is clear that the larger *T*_*3*_ is, the less possible that a false line would be wrongly detected. While on the other hand, if this ratio is too large, less lines could be detected especially if the longest line in the image are very long. For 45 sonograms of biceps and forearm, when we tune *T*_*3*_ from 10% up to 50%, all detected lines were valid but the number of total detected lines decreased from 314 to 118.

As for *T*_*1*_ and *T*_*2*_, which are thresholds for aspect ratio *Ar* and width *ω* respectively, taking an incoming region as an example, the larger its *Ar* and the smaller its *ω* is, the more possible it will falls into RA group. Simply speaking, *T*_*1*_ and *T*_*2*_ affect the number of lines detected using HT. The larger *T*_*1*_ and the smaller *T*_*2*_ is, the more lines will be detected using HT, and the slower the algorithm will be. However, smaller *T*_*1*_ or larger *T*_*2*_ could cause higher risks to mis-classify a RB as a RA, which may lead to a wrong angle estimation. Therefore, one has to compromise between the computation efficiency and correctness and tune the parameters based on the specific image contents.

### Evaluation of the validity and accuracy of the proposed method

As for the quantitative performance of our method, taking the result on the dataset used in previous study [3,5] as an example, a very good linear correlation between the results of the manual and proposed methods was obtained (shown in Figure 6, R^2^ = 0.987). Meanwhile, Bland-Altman plots in Figure 7 and Figure 8 also echoed the good agreement between the results of the manual and proposed methods both in the previous dataset and sonograms of gastrocnemius from normal subjects.

As shown in Table 1, the angle differences between the manual and automatic measurements, 0.11 ± 1.80 at the 95% confidence level, are comparable to the previous results of 0.13 ± 1.79 obtained using RVHT with Gabor filtering [5].

Results of the proposed method for interested MFO tracking agreed well with those of the manual method, as shown in Figure 9. It can be observed in Figure 10 that the interested MFOs detected were not as smooth as the torque signal, but followed the general cyclic changes along with the torque output.

The results from the aged subject with cerebral infarction have given us some preliminary confidence on the proposed framework. However, the proposed method failed to detect all valid major muscle fibers (about 7.5% missed). This could be related to the fact that the images from this subject are quite different from young or healthy subjects in terms of both echo intensity and image texture [34].

### Evaluation of the efficiency of the proposed method

Because MVEF has less computation than Gabor Filtering [20], we compared speed of our framework with that of RVHT both with and without MVEF procedure.

It can be seen from Table 2, that in general, the speed of the proposed framework is much faster than that of RVHT, which makes significant difference in terms of massive data.

Two major reasons contributed to the higher efficiency of the proposed method: Firstly, the new framework skipped the voting step as much as possible. Instead, the shape properties of muscle fibers were used to output the MFO in most cases. The time complexity for computing the orientation of the ellipse that has the same normalized second central moments of the region is *O*(n^2^), which is smaller than Hough/Radon transform for line detection in an image with the time complexity *O*(n^3^). Subsequently, the time complexity of the whole framework is related to the shape of fibers. Taking the result on the dataset used in previous study [3,5] as an example, only a very few regions are branch-structured and therefore detected using HT in sonograms. Experiments showed that the number of lines detected using HT was only 2.5% of the total number of detected lines for the 45 sonograms. As for the 230 (30 from 10 subjects and a sequence of 200 frames from the representative subject) sonograms of gastrocnemius from normal subjects, only one line was detected using HT. In other words, in most cases our framework detects lines without the voting procedures, and HT remains only a complementary operation for branch-structured fiber.

Secondly, in our framework, candidates in each voting step were pixels of a sub-region, which is different from the previous RVHT where all pixels in the image would be involved in a voting step.

Improving computation efficiency contributes a lot to the real-time application of ultrasonic diagnosis. The benefit of improved computation speed will grow much larger when we are shifting today from dealing with individual scans to several stream of video captured during the scan procedures. The high efficiency of the method is crucial for applications in the evaluation of the muscle dynamic behavior using sonograms where massive data are involved (such as [4,6-12]).

## Conclusions

In this paper we proposed a new framework for the efficient estimation of MFO using ultrasonography. The framework aimed to output angles using the shape properties of muscle fibers in sonograms with as less as possible Hough/Radon voting procedures. Results of the experiments suggest that, without compromising the accuracy, the proposed framework achieved higher efficiency than previous RVHT algorithm at line angle estimation, which is much more outstanding when massive data would be involved. Therefore, our new method would facilitate image-guided ultrasound musculoskeletal analysis better, from automatic estimation of MFO to subsequent muscle fiber length and thickness.

## Abbreviations

MFO: Muscle fiber orientation; HT: Hough transform; RVHT: Revoting hough transform; MVEF: Multiscale vessel enhancement filtering; MVC: Maximal voluntary contraction; EMG: Electromyography; RMS: Root mean square.

## Competing interests

The authors declare that they have no competing interests.

## Authors’ contributions

SL: composed the manuscript together with BC and YJZ, YJZ: proposed the idea, BC: analyzed the data with SL, YQZ, LW, WZY and YPZ: performed experiments together with YJZ. All authors read and approved the final manuscript.
